# Time‐dependent association between prenatal hair glucocorticoid levels and child behavior problems

**DOI:** 10.1111/jcpp.70131

**Published:** 2026-02-25

**Authors:** Richard G. Künzel, Yinxian Chen, Marta B. Rondon, Diana Juvinao‐Quintero, Laramie E. Duncan, Sixto E. Sanchez, Luz G. Nateros, Archana Basu, Amantia Ametaj, Clemens Kirschbaum, Elizabeth J. Levey, Bizu Gelaye

**Affiliations:** ^1^ Harvard T.H. Chan School of Public Health Boston MA USA; ^2^ Technische Universität Dresden Dresden Germany; ^3^ Department of Epidemiology Rollins School of Public Health, Emory University Atlanta GA USA; ^4^ Universidad Peruana Cayetano Heredia San Martín de Porres Peru; ^5^ Facultad de Medicina Humana, Instituto de Investigación Universidad de San Martin de Porres La Molina Peru; ^6^ Department of Psychiatry and Behavioral Sciences Stanford University Stanford CA USA; ^7^ Asociación Civil Proyectos en Salud Lima Peru; ^8^ Massachusetts General Hospital Boston MA USA; ^9^ Northeastern University Boston MA USA; ^10^ The Chester M. Pierce M.D. Division of Global Psychiatry, Department of Psychiatry Massachusetts General Hospital Boston MA USA; ^11^ Epidemiology Branch, Division of Population Health Research, Division of Intramural Research Eunice Kennedy Shriver National Institute of Child Health and Human Development Bethesda MD USA

**Keywords:** Prenatal, hair cortisol, hair cortisone, internalizing, externalizing, behavior, transgenerational

## Abstract

**Background:**

Child internalizing and externalizing behavioral problems are highly prevalent psychiatric symptoms worldwide, for which maternal prenatal stress is a known risk factor. However, underlying neuroendocrine mechanisms remain largely unclear. We investigated whether maternal hair cortisol (HCC) and cortisone concentration (HCNC) are associated with offspring's internalizing and externalizing behavior problems in a prospective pre‐birth cohort study from Perú.

**Methods:**

*N* = 271 mother–child dyads were included in this analysis. Recruitment and data collection took place at the *Instituto Nacional Materno Perinatal* in Lima, Perú. HCC and HCHC were obtained from hair segments representing up to 3 months pre‐pregnancy and first trimester, respectively, and were quantified via liquid chromatography tandem mass spectrometry. The Child Behavior Checklist was used to assess internalizing and externalizing behavioral problems of children (mean age at follow‐up = 6.98 years (*SD* = 1.05)). Marginal structural models estimated population average associations between HCC, HCNC, and internalizing and externalizing problems, adjusting for established covariates.

**Results:**

At pre‐pregnancy, logHCNC was positively associated with offspring internalizing (*β* = 2.21, 95% CI: 0.46; 3.96, *p* = .013) and externalizing problems (*β* = 1.87, 95% CI: 0.34; 3.40, *p* = .016). At the first trimester, logHCNC was negatively associated with internalizing (*β* = −2.51, 95%CI: −4.37; −0.64, *p* = .008), and externalizing problems (*β* = −2.73, 95% CI: −4.18; −1.28, *p* < .001). Associations were stronger for females and not apparent for logHCC.

**Conclusions:**

We found time‐dependent associations between stress‐related prenatal hair glucocorticoid concentration and offspring behavioral problems. Modeling biomarker data time‐dependently may prove critical to identifying the underlying mechanisms of transgenerational stress transmission.

## Introduction

Childhood is a time of continuous change and development, essential preparation for the challenges of life. Yet, these developments can themselves become challenging, often manifesting in problematic child behavior. Child behavioral problems are traditionally distinguished into internalizing and externalizing behavior, which are characterized by an inwardly‐focused over‐control and an outwardly‐focused under‐control of emotion expression, respectively (Achenbach, 1966; Nikstat & Riemann, 2020). Although moderate expressions of these behaviors are considered “developmentally normative” (Kjeldsen, Nes, Sanson, Ystrom, & Karevold, 2021), stronger expressions often manifest in neurodevelopmental conditions, such as depressive disorders or oppositional defiant disorder (APA, 2013). Overall, neurodevelopmental conditions are highly prevalent among children worldwide, with a global prevalence of approximately 13.4% (see Polanczyk, Salum, Sugaya, Caye, & Rohde, 2015). Additionally, child behavior problems are risk factors for adult psychiatric conditions (Kessler et al., 2005; see Loth, Drabick, Leibenluft, & Hulvershorn, 2014; Miettunen et al., 2014) and poor economic and social outcomes (Vergunst et al., 2023). Modest genetic influences on internalizing and externalizing behavior are well established (Nikstat & Riemann, 2020), and yet the etiology of these behavior problems remains largely unclear (Kauten & Barry, 2020). Several theoretical models, like the Developmental Origins of Health and Disease (DOHaD), which propose that in‐utero exposure to environmental factors can affect the offspring's health (see Barker, 1998; see Howland, Sandman, & Glynn, 2017), have identified prenatal maternal stress as a potentially causal risk factor (Dachew, Heron, & Alati, 2023; Kallas et al., 2023; Kang et al., 2021; Kingston, Heaman, Fell, Dzakpasu, & Chalmers, 2012; Van den Bergh et al., 2020). This raises the question of how stress regulation may be transmitted biologically in pregnancy, separate from behavioral transmission during the postpartum period.

One proposed biological system involved in this transmission of maternal to offspring stress regulation is the hypothalamic–pituitary–adrenal (HPA) axis (Dachew et al., 2023; see Tien, Lewis, & Liu, 2020), which acts through a cascade of neuroendocrine secretions culminating in the secretion of the hormone cortisol (see Sheng et al., 2021). Traditionally, cortisol has been measured via blood, urine, or saliva, but given strong situational and diurnal variations, these specimens are limited in their ability to depict humoral responses to long‐term or severe stressful experiences (see Stalder & Kirschbaum, 2012). Addressing this limitation, cortisol measurement in scalp hair has been developed as a reliable and valid method of retrospective, continuous cortisol measurement (Short et al., 2016; see Stalder & Kirschbaum, 2012). In addition to regulating stress, cortisol concentration also increases during pregnancy progressively, up to twofold, until delivery (Jung et al., 2011; King, Humphreys, Cole, & Gotlib, 2022), although in a non‐linear manner and with notable within‐person variations (Marceau, Rolan, Robertson, Wang, & Shirtcliff, 2021). Some cortisol increase appears to be necessary to support fetal brain development (see Bird, McDougall, Seow, Hooper, & Cole, 2015; Liggins, 1994). Despite fetal protection from cortisol overexposure by the enzyme 11β‐Hydroxysteroid dehydrogenase type 2 (11β‐HSD2), which converts cortisol into its biologically inactive form cortisone (see Seckl & Meaney, 2004), approximately 10%–20% of maternal cortisol crosses the placenta (see Cottrell, Seckl, Holmes, & Wyrwoll, 2014). Notably, previous studies have shown that the functioning of 11β‐HSD‐2 can be altered in the presence of maternal stress, potentially allowing stress‐related glucocorticoid secretion to directly affect fetal neurodevelopment (see Janssen et al., 2016).

Despite these links, only one previous study investigated the relation between prenatal maternal hair cortisol concentration (HCC) and child behavior (Mustonen et al., 2024). Mustonen et al. found that mid/late‐pregnancy HCC was negatively associated with total behavioral problems and internalizing problems in offspring at 2 years; early/mid‐pregnancy HCC was negatively associated with emotional problems in offspring at 5 years (2024). Although these findings provide a valuable starting point for a better understanding of the role of maternal prenatal glucocorticoids in the development of offspring behavioral problems, substantial gaps remain. For instance, it remains unclear whether biological stress transmission is primarily related to stress levels during pregnancy or whether maternal stress before pregnancy contributes as well. Prior research has indicated that traumatic stress exposure can be associated with long‐term dysregulation of cortisol secretion (see Schindler‐Gmelch, Capito, Steudte‐Schmiedgen, Kirschbaum, & Berking, 2023), which may potentially influence baseline cortisol levels up to the time of conception. Time‐differential analyses of hormone regulation around the time of conception may help to clarify whether stress‐related alterations in baseline levels before conception are reflected in hormone levels after conception and, in turn, may be implicated in fetal development. Furthermore, prior studies recommend the additional investigation of cortisone, which may be a more robust biomarker of HPA axis functioning, due to its higher concentration and a reduced susceptibility to environmental contamination compared to cortisol (Stalder et al., 2013; see Stalder & Kirschbaum, 2012; Staufenbiel, Penninx, de Rijke, van den Akker, & van Rossum, 2015). Lastly, investigations of samples from disproportionately underrepresented countries in the global south (see Marceau, Wang, Robertson, & Shirtcliff, 2020) are needed to enhance the ethnic, racial, and geographic diversity of published evidence on long‐term consequences of stress exposure, thereby helping to improve the global generalizability of scientific findings in this field. The present study contributes to this effort by providing data from Peruvian mother–child dyads, an under‐represented Latin American population particularly affected by psychosocial stressors, such as trauma exposure (Gelaye et al., 2017). In the present study, we aimed to evaluate whether prenatal maternal hair glucocorticoid concentration is associated with offspring behavioral problems.

## Methods and materials

### Study population, recruitment, and study procedure

The participants for this study were drawn from the *Pregnancy Outcomes, Maternal and Infant Study* (PrOMIS), a longitudinal pre‐birth cohort study based in Lima, Perú. Recruitment took place between February 2012 and June 2014 at the *Instituto Nacional Materno Perinatal* (INMP), the most important national reference center for reproductive care of the Peruvian Ministry of Health (Barrios et al., 2015). Eligibility criteria were initiation of prenatal care before gestational week 16, age ≥ 18 years, singleton pregnancy, Spanish language fluency, and intent to give birth at the INMP (Barrios et al., 2015). Exclusion criteria were a medical history of intellectual disability, psychosis, pre‐gestational chronic hypertension, diabetes mellitus, sepsis, antiphospholipid syndrome, and renal diseases, which were not included in the study (Barrios et al., 2015; Levey et al., 2018).

A total of *n* = 1,055 participants provided baseline information and hair samples between weeks 7 and 18 of gestation and were followed until delivery (Barrios et al., 2015). Follow‐up assessment began in 2020 and included *n* = 276 mother–child dyads until today. Among those, a total of *n* = 271 dyads with complete data were included in the analysis. All participants provided written informed consent. The study protocol was approved by the INMP and Harvard T.H. Chan School of Public Health review boards.

### Maternal and offspring's sociodemographic, anthropometric, behavioral, and medical information

During all assessments, data were collected by trained research personnel using a structured interview. The maternal sociodemographic and anthropometric data included age (in years), ethnicity (mestizo/ not mestizo), difficulty accessing basic items, such as foods (yes/ no), number of previous pregnancies (parity), and self‐reported body mass index (BMI) before pregnancy (in kg/m^2^). At the first prenatal care visit, and after interviews were completed, body weight and height were measured to calculate BMI during pregnancy as described previously (Natamba, Sanchez, Gelaye, & Williams, 2016). Pregnancy information was obtained from medical records and included gestational age at enrollment (calculated by last menstrual period, verified by ultrasound; in weeks) and fetal sex (male/female). Serum C‐reactive protein (pg/mg) was measured from maternal non‐fasting blood samples at the first assessment. The details of the measurement have been described elsewhere (Yang et al., 2017). Child behavior was assessed using the validated Spanish version of the Child Behavior Checklist (CBCL), which includes 120 problem items rated on a 3‐point scale (0 = not true, 1 = somewhat true, 2 = very true) (Achenbach, 1991; Rubio‐Stipec, Bird, Canino, & Gould, 1990). Items are grouped into seven syndrome scales for children aged 1.5–5 years and eight syndrome scales for those aged 6–18 and synthesized into two higher‐order factors: internalizing and externalizing problems. Raw scores are T‐scale transformed based on validated standard tables with the software “ASEBA‐PC” (ASEBA, 2019). Higher scores indicate more problematic behavior. The CBCL has demonstrated high validity and reliability (Albores‐Gallo et al., 2007; Ivanova et al., 2007; Rubio‐Stipec et al., 1990), and had a standardized Cronbach's Alpha of *α* = .93 in our study.

### Hair collection and glucocorticoid analysis

Hair cortisol (HCC) and hair cortisone concentration (HCNC) were obtained from maternal scalp hair samples at the end of the first trimester. A full‐length hair strand was cut at the posterior vertex of the scalp as close to the scalp as possible. Hair strands were then cut into 3 cm segments that represent a retrospective time period of up to 3 months, respectively, given an average hair growth of approximately 1 cm per month (see Pragst & Balikova, 2006; Wennig, 2000). According to previous recommendations, only the two most proximal 3‐cm hair segments were used for the analysis (Orta et al., 2018). Given the hair collection around the end of the first trimester, these two hair segments represent glucocorticoid secretion predominantly during the first trimester (0–3 cm) and the pre‐pregnancy period (3–6 cm). HCC and HCNC were obtained via liquid chromatography tandem mass spectrometry at the Dresden University of Technology with a lower detection limit of 0.1 pg/mg. Details of the laboratory procedure have been reported elsewhere (Orta et al., 2018).

### Data analysis

Statistical analyses were conducted in R, version 4.4.1 (R Core Team, 2024). HCC and HCNC were right‐skewed and thus log‐transformed and standardized. Missingness within variables was <10% for all included variables, except pre‐pregnancy body mass index (BMI; 13.6%), which was imputed using the mean sample BMI difference if the corresponding pregnancy value was available. All analyses were conducted with complete data.

Descriptive analyses included frequency, percentages, means, and standard deviations (SD). Differences in baseline characteristics between dyads with and without clinical behavioral outcome values (*T*‐scores >64) were tested using the Wilcoxon rank sum test, Pearson's Chi‐squared test, and Fisher's exact test.

Marginal structural models (MSM) examined the population average effect of HCC and HCNC as time‐varying exposures on internalizing and externalizing behavior problems (*T*‐score). Four generalized propensity scores (GPS) were generated for HCC and HCNC using the Gaussian generalized linear model to account for the anticipated influence of covariates. Covariates were selected using two strategies: identifying those significantly associated with exposure and outcome in preliminary correlation analyses (leading to pre‐pregnancy and pregnancy BMI (used in the pre‐pregnancy or the first trimester models, respectively)) and based on prior evidence (leading to maternal age, mestizo ethnicity, difficulty accessing basic items, parity, gestational age at enrollment and infant sex (in first trimester models only)) (Juvinao‐Quintero et al., 2024). Stabilized inverse‐probability weights (sIPW) were calculated based on GPS and used to fit MSMs. Diagnostics included inspecting sIPW distribution and comparing unweighted and weighted correlations between hair glucocorticoid concentrations and each included covariate. Secondary analyses included stratified MSMs fitted separately in males and females and pooled MSMs including sex × exposure interaction terms. In sensitivity analyses, linear regression analyses were used to inspect the conditional association between HCC and HCNC and internalizing and externalizing behavior problems (*T*‐scores) at each time point independently (pre‐pregnancy and first trimester) and to check for homoscedasticity and low generalized variance inflation (GVIF) across all selected covariates. Additionally, MSMs were adjusted for serum C‐reactive protein concentration in first‐trimester models (to account for potential inflammatory influences on the association; see Coussons‐Read, 2013; see Stalder & Kirschbaum, 2012). All statistical tests were two‐sided (*p* < .05), and confidence intervals were calculated at the 95% level.

## Results

### Descriptive statistics

This study included a total of 271 mother–child dyads, with complete data of *n* = 267 in HCC, and *n* = 235 in HCNC. The sociodemographic characteristics of the dyads are presented in Table 1. No substantial sociodemographic differences were observed between dyads with and without clinically significant child behavioral problems (Table S1), between the offspring sexes, or between the total number of baseline participants and the analytic sample. Glucocorticoid measurements were moderately to strongly positively correlated across subsequent timepoints and between HCC and HCNC within each timepoint (Table S2).

**Table 1:** Baseline characteristics of mother–child dyads (*N* = 271)

Characteristics	Mean/*n*	*SD*/%
Sociodemographic variables		
Maternal age (years)	27.46	6.22
Maternal age categorical		
18–20	21	7.75
21–29	154	57.20
30–34	59	21.77
≥35	36	13.28
Ethnicity Mestizo	232	85.61
Difficulty accessing basic foods, yes	126	46.67
Pre‐Pregnancy BMI (kg/m^2^)	25.34	4.29
Pre‐Pregnancy BMI categorical		
<18.5	6	2.22
18.5–24.9	135	50.00
25.0–29.9	93	34.44
≥ 30	36	13.33
Pregnancy BMI (at enrollment; kg/m^2^)	25.57	4.18
Pregnancy BMI (at enrollment; categorical)		
<18.5	7	2.59
18.5–24.9	123	45.56
25.0–29.9	106	39.26
≥30	34	12.59
Pregnancy Characteristics		
Gestational age at enrollment/ hair collection (weeks)	11.86	3.41
Parity		
0	129	47.60
1	86	31.73
≥2	56	20.66
Infant sex, Male	149	54.98
Offspring Characteristics		
Child age at assessment in years	6.98	1.05
Child age at assessment in years, categorical		
≤7	67	24.74
8	123	45.39
≥9	81	29.89
Behavioral Problems		
Externalizing problems, *n* (T > 64)	33	12.18
Internalizing problems, *n* (T > 64)^a^	45	16.61
Hair Glucocorticoid Concentration in pg/mg		
Pre‐pregnancy HCC	3.75	3.51
First trimester HCC	4.21	3.81
Pre‐pregnancy HCNC	4.50	3.82
First trimester HCNC	7.32	5.40

^a^ The clinical cut‐off value for internalizing behavior for boys of age 6–18 years is *T* = 65. Numbers may not add up to the full sample size or 100% due to missing data.

### Primary analyses

**Figure 1:**
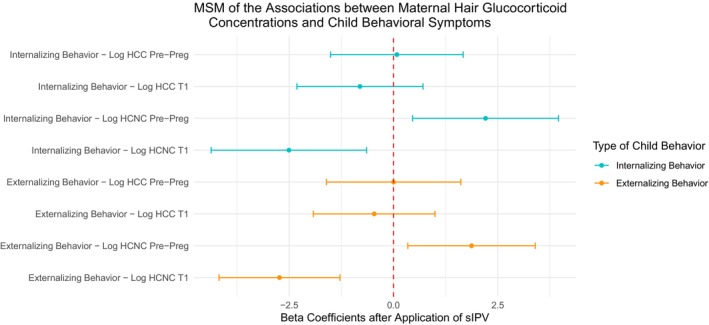
Results of marginal structural models of the associations between maternal hair glucocorticoid concentrations and child behavioral outcomes. Log HCC, log hair cortisol concentration. Log HCNC, log hair cortisone concentration. LogHCC and logHCNC have been standardized (mean = 0 and SD = 1). T1, first trimester. Pre‐Preg., pre‐pregnancy. sIPV, stabilized inverse‐probability weights. Via sIPW, associations were adjusted for pre‐pregnancy BMI (at pre‐pregnancy only), pregnancy BMI (at first trimester only), maternal age, mestizo ethnicity, difficulty paying for basic foods, parity, gestational age at enrollment and infant sex (at first trimester only)

Figure 1 and Table 2 show the results of the MSMs, which were used to estimate the population average associations between hair glucocorticoid concentrations at two occasions (pre‐pregnancy and first trimester) and offspring behavioral problems.

**Table 2:** Results of marginal structural models of the associations between maternal hair glucocorticoid concentrations and child behavioral outcomes

		Internalizing behavior		Externalizing behavior	
		*β*	95% CI	*β*	95% CI
Log HCC^a^	Pre‐pregnancy	0.08	−1.51; 1.67	0.003	−1.61; 1.61
	First Trimester	−0.80	−2.31; 0.71	−0.46	−1.92; 1.00
Log HCNC^b^	Pre‐pregnancy	2.21*	0.46; 3.96	1.87*	0.34; 3.40
	First Trimester	−2.51**	−4.37; −0.64	−2.73***	−4.18; −1.28

Log HCC, log hair cortisol concentration; Log HCNC, log hair cortisone concentration. LogHCC and logHCNC have been standardized (mean = 0 and SD = 1). Via sIPWs, associations were adjusted for pre‐pregnancy BMI (at pre‐pregnancy only), pregnancy BMI (at first trimester only), maternal age, mestizo ethnicity, difficulty paying for basic foods, parity, gestational age at enrollment and infant sex (at first trimester only).

^a^ *n* = 267.

^b^ *n* = 235.

* *p <* .05.

** *p <* .01.

*** *p <* .001.

For internalizing behavior, no significant association with logHCC was found at either time. However, regarding logHCNC, we found that a one SD increase in pre‐pregnancy logHCNC from the population mean was associated with a 2.21‐point increase in offspring internalizing behavior (*β* = 2.21, *p* = .013). In contrast, for the first trimester, we found that a one SD increase in first‐trimester logHCNC from the population mean was associated with a 2.51‐point decrease in offspring internalizing behavior (*β* = −2.51, *p* = .008; Table 2).

For externalizing behavior, no significant association with logHCC was found at either time. However, regarding logHCNC, we found that a one SD increase in pre‐pregnancy logHCNC from the population mean was associated with a 1.87‐point increase in offspring externalizing behavior (*β* = 1.87, *p* = .016). In contrast, for the first trimester, we found that a one SD increase in first‐trimester logHCNC from the population mean was associated with a 2.73‐point decrease in offspring externalizing behavior (*β* = −2.73, *p* < .001; Table 2).

### Secondary analyses

To assess effect modification by offspring sex, we estimated sex‐specific effects in two ways: stratified MSMs fitted separately in males and females (Figure S1, Table S3), and a pooled MSM including sex × exposure interaction terms (Tables S4–S5). Formal tests of sex differences are based on the interaction terms in the pooled MSM.

In the stratified analyses, no significant association was found among males, but associations for females increased in magnitude compared to the full sample. For females, a one SD increase in pre‐pregnancy logHCNC was associated with higher offspring internalizing (*β* = 4.98, *p* < .01) and externalizing problems (*β* = 3.59, *p* < .001). For the first trimester, a one SD increase in logHCNC was associated with lower internalizing (*β* = −4.74, *p* < .01) and externalizing problems (*β* = −4.05, *p* < .001). No significant associations were found between logHCC and offspring behavioral problems on either occasion. Results from the pooled MSMs largely confirmed the stratified analyses: The sex × logHCNC interaction terms were statistically significant on all occasions, except for internalizing behavior in relation to first‐trimester logHCNC, indicating that the logHCNC‐offspring behavior problems associations differed by sex. In contrast, no significant sex × logHCC interaction was found (Tables S4–S5).

### Sensitivity analyses

Linear regression models inspected the conditional association of logHCC and logHCNC with offspring behavior problems at each time period independently. Regarding the first trimester, results were similar to those in the MSM, although statistical significance was not consistently found (Figure S2, Table S6). Regarding pre‐pregnancy, associations remained non‐significant (Figure S2, Table S6). In the MSMs, the additional adjustment for serum C‐reactive protein concentration in the first trimester did not change the results from the primary analyses.

### Model diagnostics

Across the linear regression models conducted in the sensitivity analyses, residuals were normally distributed and randomly scattered around zero with no discernible pattern. The GVIF did not exceed 1.70 for any included variable across models. Regarding the MSM, the means of the stabilized weights were close to 1 (HCC: mean = 0.99, SD = 0.20; HCNC: mean = 0.97, SD = 0.31). The sIPW successfully made logHCC and logHCNC independent of the included confounding variables (Table S7). The unweighted mean absolute correlation between hair glucocorticoid and confounder variables was substantially lowered after applying weights (*r*_mean_ ≤ .10).

## Discussion

We investigated whether maternal prenatal hair glucocorticoid concentration is associated with offspring behavioral problems in a sample of Peruvian mother–child dyads. We found that maternal prenatal HCNC is significantly associated with both internalizing and externalizing behavior problems, although in temporally different manners. Whereas at pre‐pregnancy, HCNC was positively associated with behavior problems, we found negative associations between HCNC and offspring behavior problems in the first trimester. Secondary analyses revealed stronger associations among females than males.

The association between prenatal environmental exposures and offspring health is well established, as evidenced by various empirical findings within theoretical frameworks like the DOHaD (see Barker, 1998; see Howland et al., 2017). In this framework, positive associations between maternal prenatal stress and offspring behavioral problems have consistently been reported (Dachew et al., 2023; Kallas et al., 2023; Kingston et al., 2018; see McLean, Cobham, & Simcock, 2018; Van den Bergh et al., 2020), but the underlying biological mechanisms are not well understood. Our findings indicate that a prenatal HPA axis dysregulation, specifically lower glucocorticoid concentration in the first trimester of pregnancy, may play a role in these transgenerational effects of maternal stress on offspring's behavioral problems. This finding is partly in accordance with results from a previous study by Mustonen et al. (2024), who reported negative associations between HCC in mid/late pregnancy and offspring internalizing and total behavior problems at 2 years. Associations between early/mid pregnancy HCC and offspring behavior problems were similar in direction, but not statistically significant (2024). These findings are psychophysiologically plausible. Glucocorticoid concentration can be dysregulated in response to chronic or severe stress, such as after traumatic experiences (see Schindler‐Gmelch et al., 2023). Additionally, glucocorticoid concentration undergoes an average increase across pregnancy (King et al., 2022; Marceau et al., 2021) to support fetal growth, including brain development (see Matthews, 2000; see Moisiadis & Matthews, 2014). Therefore, a stress‐related dysregulation of glucocorticoid concentrations during pregnancy may limit fetal development, which can result in adverse maternal and offspring health outcomes. While the increase of maternal glucocorticoids is steeper toward the end of pregnancy (D'Anna‐Hernandez, Ross, Natvig, & Laudenslager, 2011; Jung et al., 2011; Juvinao‐Quintero et al., 2024; King et al., 2022; Marceau et al., 2021), our results indicate that glucocorticoid concentration may impact human development already early in pregnancy, provided that causality of this association can be verified in future studies.

Nevertheless, blunted increases in the first trimester may not necessarily be the only driver of adverse effects of stress‐related prenatal glucocorticoid concentrations on offspring behavioral problems, as we additionally found a positive association with offspring behavioral problems at pre‐pregnancy. This opposite direction of association from the first trimester may point to the importance of the relative change between pre‐pregnancy and first trimester glucocorticoid concentration for the development of the offspring. Presumably, women with stress‐related high glucocorticoid concentration before pregnancy are limited to undergo the natural pregnancy‐related glucocorticoid concentration increase in early pregnancy but can only maintain or decrease this level. This finding could only be shown in the MSMs but not the linear regression models that we examined in the sensitivity analyses, as the MSMs account for this time‐dependency of glucocorticoid concentrations while the linear models do not (Robins, Hernán, & Brumback, 2000). If this finding can be replicated in future studies, it may indicate that stress‐related HPA axis regulation before pregnancy also affects HPA axis regulation during pregnancy and, therefore, also fetal development.

Secondarily, three other findings stand out in our analyses. First, the association between maternal prenatal hair glucocorticoid concentrations and offspring behavior problems seems to be non‐specific in that these associations were similar across the behavioral problem factors (internalizing and externalizing). This is not surprising, given the high degree of comorbidity between them (Willner, Gatzke‐Kopp, & Bray, 2016). Furthermore, specific exposures are not consistently related to specific outcomes even when broad associations exist. For instance, early life adversity is an established risk factor for adult psychopathology, although a clear pattern between specific types of adversity and adult psychopathology has not been found yet (see McLaughlin, 2016). Second, the associations found in our study were exclusive to HCNC. This is likely due to a higher measurement precision, given cortisone's higher concentration and its lower susceptibility to contamination from synthetic glucocorticoids (Perogamvros, Keevil, Ray, & Trainer, 2010). Thirdly, stratified analyses by offspring sex revealed stronger associations in females, while males showed no significant associations. Previous studies suggest that this effect modification may arise from female fetuses' higher vulnerability to psychobiological stressors in early pregnancy due to different growth strategies (see Howland et al., 2017; Sandman, Glynn, & Davis, 2013). Whether these effects are merely different in magnitude or even sex‐specific needs to be investigated in future studies with a larger statistical power in both strata.

This study has some limitations. First, despite careful covariate selection, the possibility of unmeasured confounding and model misspecification remains. However, sensitivity analyses adjusting for serum C‐reactive protein did not alter results, while other factors, like maternal mood, could not be accounted for. Second, effect estimates were not corrected for multiple testing, given the relatively small analytic sample size. Third, child behavior problems were measured via maternal report, which could lead to misclassification. However, this approach is validated, reliable, and widely used (Albores‐Gallo et al., 2007). And fourth, the generalizability of our findings to other ethnic and racial groups is limited due to our unique cohort of mother–child dyads from Perú. Nevertheless, investigating this under‐represented sample contributes to enhancing the geographic diversity of published evidence in this field.

To our knowledge, this study is the first to examine and find time‐dependent associations between stress‐related maternal glucocorticoid concentration before and in early pregnancy and offspring behavioral problems in mothers in a low‐ or middle‐income setting. This finding allows for a better understanding of the biological mechanisms involved in transgenerational stress transmission, which can support the development of interventions that mitigate stress transmission and improve child behavioral health.

## Conflict of interest statement

No conflicts declared.

## Ethical considerations

All participants provided written informed consent. The study protocol was approved by the Instituto Nacional Materno Perinatal, Lima, Perú (17–19,747; 02/21/2017) and Harvard T.H. Chan School of Public Health, Boston, MA (16–1,473; 9/19/2016) review boards.

**Key points**

**What's known?**

- Childhood behavioral problems are linked across generations to maternal stress during pregnancy; maternal glucocorticoid levels are a potential biological pathway.

**What's new?**

- Timing matters: We show that higher stress‐related glucocorticoid levels before pregnancy, but lower stress‐related glucocorticoid levels in the first trimester, are linked with child behavioral problems, particularly among girls.
- Investigating a mother–child dyad from Peru, we add critical evidence from an understudied low‐ and middle‐income population.

**What's relevant?**

- Future research should model biomarker data with temporal sensitivity to better understand the underlying mechanisms of transgenerational stress transmission.
- If causal and replicable, our findings suggest that monitoring and supporting maternal stress regulation before and early in pregnancy may help prevent and mitigate childhood behavioral problems.

## Supporting information


**Table S1.** Differences in baseline sociodemographic variables between dyads with and without clinically significant child behavioral problems.
**Table S2.** Pearson correlations between glucocorticoid measurements.
**Table S3.** Results of linear regression analyses of the associations between maternal hair glucocorticoid concentrations and child behavioral outcomes in strata of offspring sex.
**Table S4.** Results of marginal structural models of the associations between maternal hair glucocorticoid concentrations and child behavioral outcomes in strata of offspring sex.
**Table S5.** Results of linear regression analyses of the associations between maternal hair glucocorticoid concentrations and child behavioral outcomes.
**Table S6.** Average absolute correlations between hair glucocorticoid concentrations and the covariate with and without stabilized inverse‐probability weighting.
**Table S7.** Average absolute correlations between hair glucocorticoid concentrations and the covariate with and without stabilized inverse‐probability weighting.
**Figure S1.** Results of adjusted linear regression analyses of the associations between maternal hair glucocorticoid concentrations and child behavioral outcomes in strata of offspring sex.
**Figure S2.** Results of marginal structural models of the associations between maternal hair glucocorticoid concentrations and child behavioral outcomes in strata of offspring sex.

## Data Availability

The data that support the findings of this study are available on request from the corresponding author. The data are not publicly available due to privacy or ethical restrictions.
