# Supplementary material for: Time‐dependent association between prenatal hair glucocorticoid levels and child behavior problems
Source: J Child Psychol Psychiatry. 2026 Feb 25;67(8):1297–306. doi: 10.1111/jcpp.70131 (PMC13188062; doi:10.1111/jcpp.70131)
Supplement: Supplementary file 1 — Table S1. Differences in baseline sociodemographic variables between dyads with and without clinically significant child behavioral problems. Table S2. Pearson correlations between glucocorticoid measurements. Table S3. Results of linear regression analyses of the associations between maternal hair glucocorticoid concentrations and child behavioral outcomes in strata of offspring sex. Table S4. Results of marginal structural models of the associations between maternal hair glucocorticoid concentrations and child behavioral outcomes in strata of offspring sex. Table S5. Results of linear regression analyses of the associations between maternal hair glucocorticoid concentrations and child behavioral outcomes. Table S6. Average absolute correlations between hair glucocorticoid concentrations and the covariate with and without stabilized inverse‐probability weighting. Table S7. Average absolute correlations between hair glucocorticoid concentrations and the covariate with and without stabilized inverse‐probability weighting. Figure S1. Results of adjusted linear regression analyses of the associations between maternal hair glucocorticoid concentrations and child behavioral outcomes in strata of offspring sex. Figure S2. Results of marginal structural models of the associations between maternal hair glucocorticoid concentrations and child behavioral outcomes in strata of offspring sex. [file JCPP-67-1297-s001.docx]

**Time-Dependent Association Between Prenatal Hair Glucocorticoid Levels and Child Behavior Problems**

# Supporting Information

**Table S1.** Differences in baseline sociodemographic variables dyads with and without clinically significant child behavioral problems.

**Table S2.** Pearson correlations between glucocorticoid measurements.

**Figure S1**. Results of marginal structural models of the associations between maternal hair glucocorticoid concentrations at two occasions and child behavioral outcomes within strata of offspring sex.

**Table S3.** Results of marginal structural models of the associations between maternal hair glucocorticoid concentrations at two occasions and child behavioral outcomes within strata of offspring sex.

**Table S4.** Results of marginal structural models of the associations between maternal hair cortisol concentrations (HCC) at two occasions and child behavioral outcomes with interaction terms for offspring sex (*n* = 267)

**Table S5.** Results of marginal structural models of the associations between maternal hair cortisone concentrations (HCNC) at two occasions and child behavioral outcomes with interaction terms for offspring sex (*n* = 235).

**Figure S2**. Results of adjusted linear regression analyses of the associations between maternal hair glucocorticoid concentrations and child behavioral outcomes.

**Table S6.** Results of adjusted linear regression analyses of the associations between maternal hair glucocorticoid concentrations and child behavioral outcomes.

**Table S7.** Average absolute correlations between hair glucocorticoid concentrations and the covariate with and without stabilized inverse probability weighting

**Table S1.** Differences in baseline sociodemographic variables dyads with and without clinically significant child behavioral problems.

|  | **Externalizing Behavior Problems** | | | | | **Internalizing Behavior Problems** | | | | |
| --- | --- | --- | --- | --- | --- | --- | --- | --- | --- | --- |
|  | ***No* (*n* = 238)** | | ***Yes* (*n* = 33) ^a^** | |  | ***No* (*n* = 226)** | | ***Yes* (*n* = 45) ^b^** | |  |
| **Characteristics** | **Mean/ *n*** | ***SD/ %*** | **Mean/ *n*** | ***SD/ %*** | ***p-value ^c^*** | **Mean/ *n*** | ***SD/ %*** | **Mean/ *n*** | ***SD/ %*** | ***p-value ^c^*** |
| Sociodemographic Variables |  |  |  |  |  |  |  |  |  |  |
| Maternal age (years) | 27.57 | 6.24 | 26.67 | 6.17 | 0.42 | 27.87 | 6.26 | 25.40 | 5.65 | **0.01 *** |
| Maternal age categorical |  |  |  |  | 0.89 |  |  |  |  | 0.21 |
| 18-20 | 19 | 7.98 | 2 | 6.06 |  | 18 | 7.97 | 3 | 6.67 |  |
| 21-29 | 134 | 56.30 | 21 | 63.64 |  | 123 | 54.42 | 33 | 71.11 |  |
| 30-34 | 52 | 21.85 | 7 | 21.21 |  | 53 | 23.45 | 6 | 13.33 |  |
| ≥ 35 | 33 | 13.87 | 3 | 9.09 |  | 32 | 14.16 | 4 | 8.89 |  |
| Ethnicity Mestizo | 205 | 86.13 | 27 | 81.82 | 0.60 | 193 | 85.40 | 39 | 86.67 | 0.83 |
| Difficulty accessing  basic foods, yes | 114 | 48.10 | 12 | 36.36 | 0.21 | 104 | 46.22 | 22 | 48.89 | 0.74 |
| Pre-Pregnancy BMI (kg/m^2^) | 25.38 | 4.20 | 25.00 | 4.97 | 0.49 | 25.49 | 4.22 | 24.60 | 4.60 | 0.18 |
| Pre-Pregnancy BMI categorical |  |  |  |  |  |  |  |  |  | 0.20 |
| <18.5 | 4 | 1.68 | 2 | 6.25 | 0.36 | 3 | 1.33 | 3 | 6.67 |  |
| 18.5-24.9 | 120 | 50.42 | 15 | 46.88 |  | 114 | 50.67 | 21 | 46.67 |  |
| 25.0-29.9 | 83 | 34.87 | 10 | 31.25 |  | 77 | 34.22 | 16 | 35.56 |  |
| ≥ 30 | 31 | 13.03 | 5 | 15.63 |  | 31 | 13.78 | 5 | 11.11 |  |
| Pregnancy BMI (kg/m^2^) | 25.54 | 4.09 | 25.72 | 4.85 | 0.93 | 25.64 | 4.11 | 25.20 | 4.51 | 0.33 |
| Pregnancy BMI categorical |  |  |  |  |  |  |  |  |  | 0.83 |
| <18.5 | 6 | 2.52 | 1 | 3.13 | 0.84 | 6 | 2.67 | 1 | 2.22 |  |
| 18.5-24.9 | 107 | 44.96 | 16 | 50.00 |  | 100 | 44.44 | 23 | 51.11 |  |
| 25.0-29.9 | 95 | 39.92 | 11 | 34.38 |  | 91 | 40.44 | 15 | 33.33 |  |
| ≥ 30 | 30 | 12.61 | 4 | 12.50 |  | 28 | 12.44 | 6 | 13.33 |  |
| Pregnancy Characteristics |  |  |  |  |  |  |  |  |  |  |
| Gestational age at  enrolment (weeks) | 11.82 | 3.39 | 12.18 | 3.57 | 0.60 | 11.84 | 3.44 | 12.00 | 3.32 | 0.72 |
| Parity |  |  |  |  | 0.08 |  |  |  |  | 0.13 |
| 0 | 115 | 48.32 | 14 | 42.42 |  | 111 | 49.12 | 18 | 40.00 |  |
| 1 | 74 | 31.09 | 12 | 36.36 |  | 67 | 29.65 | 19 | 42.22 |  |
| ≥ 2 | 52 | 20.59 | 7 | 21.21 |  | 48 | 21.23 | 8 | 17.78 |  |
| Infant sex, male | 131 | 55.04 | 18 | 54.55 | 0.96 | 118 | 52.21 | 31 | 68.89 | **0.04 *** |
| Offspring Characteristics |  |  |  |  |  |  |  |  |  |  |
| Child age at assessment  in years | 6.95 | 1.06 | 7.18 | 0.98 | 0.32 | 6.98 | 1.04 | 6.96 | 1.11 | 0.97 |
| Child age at assessment  in years, categorical |  |  |  |  | 0.68 |  |  |  |  | 0.71 |
| ≤ 7 | 61 | 25.63 | 6 | 18.18 |  | 55 | 24.22 | 12 | 26.67 |  |
| 8 | 107 | 44.96 | 16 | 48.48 |  | 105 | 46.46 | 18 | 40.00 |  |
| ≥ 9 | 70 | 29.41 | 11 | 33.33 |  | 66 | 29.20 | 15 | 33.33 |  |

*Note.* ^a^ Clinical cut-off value for internalizing behavior: T = 64 (for boys aged 6-18 years: T = 65). ^b^ Clinical cut-off value for externalizing behavior: T = 64. *^c^* Based on Wilcoxon rank sum test; Fisher’s exact test and Pearson’s Chi-squared test. Numbers may not add up to the full sample size or 100% due to missing data.

**Table S2.** Pearson correlations between glucocorticoid measurements.

|  | Log first trimester HCC | Log pre-pregnancy HCNC | Log first trimester HCNC |
| --- | --- | --- | --- |
| Log pre-pregnancy HCC | .64 ^a^ | .55 ^b^ | .42 ^b^ |
| Log first trimester HCC | - | .43 ^b^ | .58 ^b^ |
| Log pre-pregnancy HCNC | - | - | .72 ^b^ |

*Note.* HCC = hair cortisol concentration; HCNC = hair cortisone concentration. ^a^ *n* = 267. ^b^ *n* = 235.

**Figure S1.** Results of marginal structural models of the associations between maternal hair glucocorticoid concentrations at two occasions and child behavioral outcomes within strata of offspring sex.

1. females
2. males

**Table S3.** Results of marginal structural models of the associations between maternal hair glucocorticoid concentrations at two occasions and child behavioral outcomes within strata of offspring sex.

|  |  | **Female offspring** | | | | | **Male offspring** | | | |
| --- | --- | --- | --- | --- | --- | --- | --- | --- | --- | --- |
|  |  | **Internalizing behavior**  (*n* = 117) | | **Externalizing behavior**  (*n* = 104) | | | **Internalizing behavior** (*n* = 147) | | **Externalizing behavior**  (*n* = 128) | |
|  |  | β | 95%CI | | β | 95%CI | β | 95%CI | β | 95%CI |
| Log HCC | Pre-pregnancy | 1.12 | -1.04; 3.29 | | -0.36 | -2.01; 2.73 | -0.50 | -2.64; 1.64 | 0.55 | -1.72; 2.82 |
|  | First Trimester | -0.63 | -2.65; 1.39 | | -0.45 | -2.82; 2.92 | -1.17 | -3.19; 0.86 | -0.94 | -2.89; 1.01 |
| Log HCNC | Pre-pregnancy | **4.95 ***** | 2.12; 7.79 | | **3.75 ***** | 1.81; 5.69 | -0.66 | -2.73; 1.41 | 0.23 | -1.75; 2.20 |
|  | First Trimester | **-4.45 ***** | -7.10; -1.80 | | **-3.94 ***** | -5.97; -1.90 | -0.81 | -3.27; 1.65 | -1.41 | -3.25; 0.44 |

*Note.* CI = 95% Confidence Interval. HCC = hair cortisol concentration. HCNC = hair cortisone concentration. Adjustment at pre-pregnancy: maternal age, ethnicity, difficulty paying for basics, married, parity, education, gestational age at enrollment, pre-pregnancy BMI. Adjustment at the first trimester: maternal age, ethnicity, difficulty paying for basics, married, parity, education, gestational age at enrollment, pregnancy BMI, infant sex. *p <* .05, ** *p <* .01, *** *p <* .001.

|  | **Internalizing behavior** | | **Externalizing behavior** | |
| --- | --- | --- | --- | --- |
|  | **β** | **95%CI** | **β** | **95%CI** |
| Log HCC Pre-pregnancy | 1.00 | -1.00; 3.01 | 0.12 | -2.08; 2.32 |
| Log HCC first trimester | -0.57 | -2.57; 1.43 | -0.28 | -2.43; 1.87 |
| Sex | **2.48 *** | 0.22; 4.74 | 0.89 | -1.17; 2.95 |
| Interaction: Sex ^a^ × Log HCC Pre-pregnancy | -1.75 | -4.71; 1.22 | -0.24 | -3.40; 2.93 |
| Interaction: Sex ^a^ × Log HCC First trimester | -0.412 | -3.30; 2.48 | -0.31 | -3.23; 2.61 |

**Table S4.** Results of marginal structural models of the associations between maternal hair cortisol concentrations (HCC) at two occasions and child behavioral outcomes with interaction terms for offspring sex (*n* = 267)

*Note.* CI = 95% Confidence Interval. HCC = hair cortisol concentration. LogHCC has been standardized (mean = 0 and SD = 1). Via sIPWs, associations were adjusted for pre-pregnancy BMI (at pre-pregnancy only), pregnancy BMI (at first trimester only), maternal age, mestizo ethnicity, difficulty paying for basic foods, parity, gestational age at enrolment and infant sex (at first trimester only). *p <* .05, ** *p <* .01, *** *p <* .001*.* ^a^ Reference value: Female.

|  | **Internalizing behavior** | | **Externalizing behavior** | |
| --- | --- | --- | --- | --- |
|  | **β** | **95%CI** | **β** | **95%CI** |
| Log HCNC Pre-pregnancy | **4.93 ***** | 2.27; 7.60 | **4.02 ***** | 2.38; 5.67 |
| Log HCNC first trimester | **-4.17 **** | -6.65; -1.68 | **-4.24 ***** | -5.94; -2.54 |
| Sex ^a^ | **2.97 *** | 0.58; 5.37 | 0.83 | -1.33; 2.99 |
| Interaction: Sex ^a^ × Log HCNC Pre-pregnancy | **-5.18 **** | -8.51; -1.85 | **-4.03 **** | -6.59; -1.48 |
| Interaction: Sex ^a^ × Log HCNC First trimester | 2.93 | -0.62; 6.48 | **2.68 *** | 0.21; 5.16 |

**Table S5.** Results of marginal structural models of the associations between maternal hair cortisone concentrations (HCNC) at two occasions and child behavioral outcomes with interaction terms for offspring sex (*n* = 235).

*Note.* CI = 95% Confidence Interval. HCNC = hair cortisone concentration. LogHCC has been standardized (mean = 0 and SD = 1). Via sIPWs, associations were adjusted for pre-pregnancy BMI (at pre-pregnancy only), pregnancy BMI (at first trimester only), maternal age, mestizo ethnicity, difficulty paying for basic foods, parity, gestational age at enrolment and infant sex (at first trimester only). *p <* .05, ** *p <* .01, *** *p <* .001*.* ^a^ Reference value: Female.

**Figure S2.** Results of adjusted linear regression analyses of the associations between maternal hair glucocorticoid concentrations and child behavioral outcomes.

*Note.* Log HCC = log hair cortisol concentration. Log HCNC = log hair cortisone concentration. Pre-Preg. = pre-pregnancy. T1 = first trimester. LogHCC and logHCNC have been standardized (mean = 0 and SD = 1). Associations were adjusted for pre-pregnancy BMI (at pre-pregnancy only), pregnancy BMI (at first trimester only), maternal age, mestizo ethnicity, difficulty paying for basic foods, parity, gestational age at enrolment and infant sex (at first trimester only).

**Table S6.** Results of adjusted linear regression analyses of the associations between maternal hair glucocorticoid concentrations and child behavioral outcomes.

|  |  | **Internalizing behavior** | | | | **Externalizing behavior** | | | |
| --- | --- | --- | --- | --- | --- | --- | --- | --- | --- |
|  |  | **Unadjusted** | | **Adjusted ^c^** | | **Unadjusted** | | **Adjusted ^c^** | |
|  |  | **β** | **95%CI** | **β** | **95%CI** | **β** | **95%CI** | **β** | **95%CI** |
| Log HCC ^a^ | Pre-pregnancy | -0.42 | -1.60; 0.73 | -0.48 | -1.60; 0.68 | -0.22 | -1.30; 0.84 | -0.42 | -1.50; 0.63 |
|  | First Trimester | -0.70 | -1.80; 0.44 | -0.81 | -2.00; 0.33 | -0.46 | -1.50; 0.59 | -0.72 | -1.80; 0.33 |
| Log HCNC ^b^ | Pre-pregnancy | 0.23 | -0.98; 1.40 | 0.10 | -1.20; 1.40 | 0.22 | -0.90; 1.30 | -0.28 | -1.40; 0.87 |
|  | First Trimester | -0.87 | -2.10; 0.35 | -1.10 | -2.30; 0.20 | -0.94 | -2.10; 0.18 | **-1.47 *** | -2.60;  -0.34 |

*Note.* Log HCC = log hair cortisol concentration. Log HCNC = log hair cortisone concentration. LogHCC and logHCNC have been standardized (mean = 0 and SD = 1). ^a^ *n* = 267. ^b^ *n* = 235. ^c^ Associations were adjusted for pre-pregnancy BMI (at pre-pregnancy only), pregnancy BMI (at first trimester only), maternal age, mestizo ethnicity, difficulty paying for basic foods, parity, gestational age at enrolment and infant sex (at first trimester only). * *p <* .05, ** *p <* .01, *** *p <* .001.

**Table S7.** Average absolute correlations between hair glucocorticoid concentrations and the covariate with and without stabilized inverse probability weighting.

|  | **Pre-pregnancy** | | **First trimester** | |
| --- | --- | --- | --- | --- |
|  | **Unweighted AAC** | **Weighted AAC** | **Unweighted AAC** | **Weighted AAC** |
| Log HCC ^a^ | 0.05 | 0.01 | 0.06 | 0.04 |
| Log HCNC ^b^ | 0.13 | 0.04 | 0.11 | 0.10 |

*Note.* AAC = average absolute correlation; HCC = hair cortisol concentration; HCNC = hair cortisone concentration. ^a^ *n* = 267. ^b^ *n* = 235.
